# Exploring the Role of Resistance Training in the Prevention and Management of Chronic Conditions: A Narrative Review

**DOI:** 10.3390/clinpract16080157

**Published:** 2026-08-21

**Authors:** Anu M. Räisänen

**Affiliations:** Office of the Provost and Academic Affairs, Elon University, Elon, NC 27244, USA; araisanen@elon.edu

**Keywords:** strength training, resistance training, physical activity, chronic disease, primary disease prevention

## Abstract

Physical activity is widely recognized as a fundamental component of health promotion and chronic disease prevention. Historically the focus has primarily been on aerobic exercise, while resistance training has received comparatively less attention. This imbalance underestimates the critical role of muscular performance in supporting physical health, functional capacity, long-term mobility, and overall quality of life. Current physical activity guidelines for Americans recommend that adults engage regularly in both aerobic and muscle-strengthening activities. However, only approximately one-quarter of adults in the United States meet these recommendations, despite nearly half achieving recommended levels of aerobic activity alone. Although any form of physical activity can confer health benefits, participation in both aerobic exercise and muscle-strengthening, such as resistance training, produces more comprehensive and substantial health outcomes. As research on resistance training has evolved beyond its traditional focus on athletic performance, a growing body of evidence has demonstrated its effectiveness in the prevention and management of numerous chronic conditions. This narrative review summarizes the role of resistance training in the prevention and management of several prevalent lifestyle-related chronic conditions, including type 2 diabetes, obesity, hypertension, cardiovascular disease, dyslipidemia, and osteoporosis.

## 1. Introduction

It has been well established that regular physical activity promotes good physical health [1]. The current physical activity guidelines for Americans recommend a combination of aerobic physical activity and muscle-strengthening activities for everyone, providing specific recommendations for children and adolescents; adults; older adults; and pregnant and postpartum women [2]. Regarding muscle-strengthening activities, the recommendation for adults and older adults is training all major muscle groups at a moderate or greater intensity at least two days a week. While health by definition is a broader context than the absence of disease [3], it is important to note that both aerobic physical activity and resistance training have an important role in the prevention and management of chronic conditions.

Lifestyle-related chronic conditions, such as heart disease and type 2 diabetes, are among the leading causes of morbidity and mortality worldwide [4,5]. While the increased burden of chronic conditions is to some extent related to population aging [6], the current evidence clearly demonstrates that these conditions have become common among people of various age, gender and socioeconomic groups, indicating that this is a long-term issue that is not purely related to demographic aging [4]. One of the challenges for individuals living with chronic conditions is that health-related quality of life is typically reduced and multimorbidity is common [7]. Due to the challenges influencing individuals and the healthcare system, reducing the burden of lifestyle-related chronic conditions through prevention and management needs be a priority for the 21st century [4].

Lifestyle-related chronic conditions are not just a result of lifestyle behaviors as the etiology is significantly influenced by genetics and other factors [8,9]. However, lifestyle choices, like physical activity participation, can impact the prevention and management of these conditions. In the field of physical activity research, the health benefits of muscle-strengthening activities have been studied less frequently than those of aerobic activity [10]. It is important to note that less people engage in muscle-strengthening compared to aerobic activity, highlighting the importance of supporting increased participation in muscle-strengthening activities. In the United States, 46.9% of adults meet the guidelines for aerobic physical activity, while 31.0% meet the guidelines for muscle-strengthening activity [11]. Notably, only 24.2% meet the guidelines for both aerobic and muscle-strengthening activities. Research has demonstrated that the greatest health benefits are yielded from the combination of both types of activity, and therefore it is important to increase knowledge on the health benefits of muscle-strengthening activities.

While muscle-strengthening can be achieved through some activities of daily living, like chopping firewood, muscular performance is typically developed through progressive resistance training. Resistance training is defined as a method of physical conditioning in which muscle contractions are used against external resistance [12]. Resistance is typically provided by free weights, machines, resistance bands or body weight, and muscle action can be isometric, isotonic or isokinetic [12]. Resistance training is further categorized into strength, hypertrophy, endurance, and power training based on the targeted adaptations [13]. While the term ‘strength training’ is often used interchangeably with ‘resistance training’, it is important to note that not all resistance training is strength training. Training for strength, hypertrophy, endurance or power adaptations is programmed differently through exercise selection and manipulating the variables of volume, intensity, velocity of muscle action, duration, frequency, and rest periods [13,14].

The purpose of this review was to summarize the benefits of resistance training, independently or in combination with aerobic activity, as an intervention to prevent and/or manage common lifestyle-related chronic conditions. This review focused on seven lifestyle-related chronic conditions frequently encountered in primary care settings: type 2 diabetes, obesity, hypertension, cardiovascular disease, dyslipidemia, and osteoporosis. It is important to note that the benefits of resistance training are not limited to these conditions. Some other relevant conditions would be sarcopenia and some common cancers, for example, but those are not covered in this narrative review.

## 2. Methods

### 2.1. Design and Rationale

This study is a narrative review that aimed to summarize evidence verbally rather than provide pooled effect estimates. Accordingly, this review was not designed as a systematic review, and it does not claim exhaustive coverage or full reproducibility.

### 2.2. Information Sources and Study Selection

Searches were conducted in PubMed for peer-reviewed articles published in English. Search terms were a combination of MeSH terms and text words of the condition names (e.g., diabetes mellitus type 2; type 2 diabetes) and resistance training (e.g., resistance training, strength training, muscle-strengthening activities). Additional information sources were articles identified through reference lists and key articles previously identified by the author, such as clinical practice guidelines. Treatment guidelines and systematic reviews with meta-analysis were prioritized. Individual trials or observational studies were included where systematic reviews or treatment guidelines were not identified, mostly for prevention. All types of resistance training were included.

## 3. Role of Resistance Training in the Prevention and Treatment of Chronic Conditions

### 3.1. Type 2 Diabetes

Type 2 diabetes is a growing global health concern and is characterized primarily by insulin resistance [15,16,17]. When muscle cells become less sensitive to insulin, their ability to absorb glucose from the bloodstream is diminished [18]. This reduced glucose uptake leads to an accumulation of glucose in the blood, causing elevated blood glucose levels [18].

Resistance training is a valuable tool in the prevention of type 2 diabetes as regular resistance training has been shown to reduce the risk of type 2 diabetes in both men [19] and women [20,21]. A cohort study reported that men who participated in resistance training demonstrated a reduced risk of type 2 diabetes compared to those with no resistance training [19]. This association was independent of aerobic activity and the risk reduction was comparable to that of aerobic exercise. The results demonstrated that even a modest amount of regular resistance training is beneficial for diabetes prevention, but the greatest benefits can be yielded from participation in aerobic activity and resistance training [19]. Similar results have been reported in large cohort studies on middle-aged and older women [20]. It is important to note that this analysis also included lower-intensity conditioning activities, such as yoga.

When it comes to the management of type 2 diabetes, meta-analytic evidence demonstrates that resistance training, independently or combined with aerobic exercise, significantly improves glycemic control in middle-aged and older adults with type 2 diabetes, producing clinically meaningful reductions in hemoglobin A_1c_ (HbA_1c_) while also improving several cardiovascular risk factors (Table 1) [22,23,24]. A meta-analysis of 29 trials comprising 1301 middle-aged or older adults with type 2 diabetes reported that high-intensity resistance training led to significant reductions in HbA_1c_ compared to usual care [23]. High intensity was defined as ≥70% 1-repetition maximum or ≤6–8 repetition maximum. A meta-analysis of four studies with a total of 261 patients demonstrated that resistance training interventions were associated with a −0.57 percentage point (95% CI −1.14 to −0.01) reduction in HbA_1c_, compared with control [24]. However, aerobic exercise was slightly more effective in reducing HbA_1c_ levels [24]. A recent meta-analysis of 100 studies with 7195 participants found that a combination of resistance training and aerobic exercise was the most effective in improving HbA_1c_, leading to a −0.74 percentage point (95% CI −0.91 to −0.57) reduction [22]. Resistance training alone resulted in a −0.36 percentage point (95% CI −0.51 to −0.20) reduction in HbA_1c_ [22].

Evidence suggests that greater improvements in muscular strength are associated with more favorable health outcomes. A meta-analysis of 20 studies comprising 1172 adults with type 2 diabetes identified a connection between improvements in muscular strength and change in HbA_1c_, reporting that the more participants improved their muscular strength, the greater the reduction in their HbA_1c_ [25].

It is important to note that some of the benefits of combined resistance training and aerobic activity can be due to the greater training dose. In some trials comparing different training interventions, the combination group performed both the aerobic and resistance training interventions, leading to approximately doubled weekly training duration [26]. However, it is important to note that a study aiming to keep weekly duration similar across the groups reported that only the combination of aerobic and resistance training led to significant improvements in HbA_1c_ [27].

In summary, resistance training has been shown to be an effective tool for the prevention of type 2 diabetes. For the management of type 2 diabetes, meta-analytic evidence is inconsistent regarding whether the greatest HbA1c reductions are achieved through high-intensity resistance training or a combination of aerobic and resistance training. Nevertheless, the evidence clearly supports resistance training as a valuable intervention for improving glycemic control.

### 3.2. Obesity

Obesity is a chronic condition characterized by excessive body fat [28]. While obesity is a serious disease influenced by genetic, epigenetic, physiological, behavioral, socio-cultural and environmental factors, it is important to note that its progression is not unavoidable [28,29]. Current obesity management guidelines and recommendations emphasize a comprehensive lifestyle approach that includes regular physical activity [28,30,31,32].

It is important to note that many studies in this section are considered relevant for both prevention and management as they include overweight participants as well as participants with obesity. Overweight participants can be considered a target for prevention of obesity while studying participants with obesity provides information to support management. However, results are typically reported for the total sample; therefore this section is not divided to separate results on prevention and management.

Resistance training is an effective tool for reducing body fat, visceral adipose tissue, subcutaneous adipose tissue, body mass and body mass index in individuals with overweight/obesity [33,34]. According to a meta-analysis of 116 studies with overweight/obese participants across the lifespan, resistance training combined with caloric restriction was the most effective intervention for reducing body fat percentage: −3.8 (95% CI −4.7 to −2.9) percentage points [33]. To reduce total body mass, resistance training with caloric restriction and resistance training combined with aerobic exercise and caloric restriction were the most effective: −5.3 (95% CI −7.2 to −3.5) kg [33]. Resistance training alone, without caloric restriction, yielded small improvements in body fat percentage [−1.6 (95% CI −1.9 to −1.2) percentage points] and total fat mass [−1.0 (95% CI −1.4 to −0.7) kg]. Similarly, a network meta-analysis in adults aged 55–70 with overweight/obesity found that resistance training alone significantly reduced total fat mass [−1.49 (95% CI −2.52 to −0.45) kg], although to a lesser extent than interventions involving energy restriction [energy restriction −4.26 (95% CI −5.35 to −3.17) kg; energy restriction + high protein −5.86 (95% CI −7.34 to −4.37) kg] [34]. A summary of the effect sizes is presented in Table 2.

In addition to reducing total body fat, body composition can be improved through increased lean body mass. Resistance training has been identified as the most effective way to increase lean body mass in children/adolescents, young adults, middle-aged adults and older adults [effect size 0.8 (95% CI 0.6 to 1.0) kg] [33]. Similarly, a network meta-analysis focusing on adults with obesity identified resistance training as the most effective exercise modality to improving lean body mass [35]. It is important to note that resistance training combined with caloric restriction did not lead to improvements in lean body mass [−0.2 (95% CI −1.2 to 0.8) kg], while it did reduce total fat mass [−5.1 (95% CI −6.3 to −3.8) kg] (Table 2) [33].

It has been reported that adults with overweight/obesity can achieve significant improvements in total fat mass and lean body mass irrespective of weekly training volume, peak intensity or resistance training duration [36]. In practical terms, this means that even lower-volume resistance programs, which tend to be better for adherence, can yield substantial benefits [36].

In addition to improving body composition, resistance training interventions can promote physical function in adults with overweight/obesity. Resistance training has been shown to improve lower and upper body strength, gait speed and the ability to perform activities of daily living in this population [37]. It is worth emphasizing that pairing resistance training with caloric restriction produced less improvement in physical function than resistance training by itself [37].

It should be noted that all the meta-analyses referenced earlier included studies with participants of different genders and sex, but there are some results indicating that sex and/or gender can influence the impact of resistance training. A meta-analysis of 20 studies including 2062 women with obesity reported that resistance training was not effective in reducing body fat percentage [38]. A comparison of results between female and male participants found that males experienced a two-fold greater reduction in fat mass compared to females as a result of resistance training [36]. Most of the studies focusing on women have included a very small sample, highlighting the need for more adequately powered intervention studies on resistance training among women.

In summary, resistance training can be a valuable tool in the prevention and management of obesity. However, interventions including caloric restriction outweigh resistance training alone when the goal is to reduce total mass or body fat percentage. It is important to consider the individual, their goals, current health status, physical functioning and body composition when selecting the ideal intervention. In some cases, focusing on increasing lean body mass or improving physical functioning through resistance training without caloric restriction can be a better goal and a more feasible intervention than aiming to reduce fat mass through caloric restriction. Lean body mass can increase resting metabolic rate and daily energy expenditure, and physical function supports functional independence and quality of life [37,39].

### 3.3. Hypertension

Hypertension refers to systemic arterial pressure consistently greater than or equal to 130 mmHg for systolic or 80 mmHg for diastolic pressure [40]. Much of the early research on the effects of resistance training on resting blood pressure largely focused on normotensive populations [41]. For hypertension management, nonpharmacological interventions have typically explored aerobic exercise [42].

However, it has been established that resistance training can be safely implemented among individuals with hypertension and the research has expanded to explore the impacts of resistance training among prehypertensive and hypertensive populations [43,44]. A 2017 meta-analysis of five studies with 201 prehypertensive and hypertensive adults reported a −8.2 (95% CI −10.9 to −5.50) mmHg reduction in systolic and −4.1 (95% CI −6.3 to −1.9) mmHg reduction in diastolic blood pressure as a result of resistance training intervention [43]. A 2023 meta-analysis of 14 studies with a total sample of 253 hypertensive adults concluded that resistance training interventions led to a decrease in both mean systolic [−9.52 (95% CI −12.89 to −6.14)] mmHg and diastolic [−5.19 (95% CI −7.98 to −2.39)] mmHg blood pressure values [44]. It was also reported that systolic blood pressure had greater sensitivity to resistance training than diastolic blood pressure [44]. The strongest effect on blood pressure was observed in studies utilizing a protocol with a moderate to vigorous load (>60% of one-repetition maximum), training frequency of at least two days/week and an intervention duration of at least 8 weeks [44]. A summary of the effect sizes is presented in Table 3.

The American College of Sports Medicine’s pronouncement on physical activity and hypertension states that aerobic and resistance training alone or combined are equally effective in lowering blood pressure in normotensive, prehypertensive and hypertensive individuals [45]. There has been hesitancy in prescribing resistance training to individuals with hypertension due to the hemodynamic response [46]. However, current guidelines state that the benefits of physical activity outweigh the risks [1,46]. It is still important to note that very high resting blood pressure is a contraindication to exercise and an excessive blood pressure response to exercise is an indication to terminate exercise [46]. In summary, resistance training is a suitable tool to be used with prehypertensive and hypertensive adults and older adults to prevent and manage hypertension. Prehypertensive and hypertensive individuals should consult with their doctor regarding specific guidelines to follow for safe physical activity participation.

### 3.4. Cardiovascular Disease

Cardiovascular disease is a collective term for a group of disorders of the heart and blood vessels, including but not limited to coronary artery disease and heart failure [47]. Cardiovascular diseases are the leading cause of death globally [4,5].

The American Heart Association recently updated their scientific statement regarding resistance training and cardiovascular disease to reflect the current evidence [48]. The statement includes recommendations for resistance training prescription and provides examples of exercises for the major muscle groups [48]. The statement recognizes that resistance training is important in the prevention of cardiovascular disease [48]. The importance of resistance training in the prevention of cardiovascular disease is supported also by a meta-analysis, which reported that adults who participate in muscle-strengthening activities have a 17% lower risk of developing cardiovascular disease, compared to those who report no muscle-strengthening activities [10].

In addition to primary prevention, resistance training also has an important role in the management of different cardiovascular diseases. For coronary artery disease, it has been established that cardiorespiratory fitness can predict mortality among individuals with coronary artery disease and therefore is a key outcome in disease management [49]. Traditionally exercise-based cardiovascular rehabilitation has focused on aerobic exercise but recent research has reported that adding resistance training can lead to better outcomes [50]. A recent meta-analysis of 23 studies with 916 participants with coronary artery diseases compared combined aerobic and resistance training to aerobic training alone [50]. The results demonstrated that combined training was more effective in increasing cardiorespiratory fitness [mean difference 0.26 (95% CI 0.02 to 0.49)] and lean body mass [0.8 (95% CI 0.4 to 1.2) kg] and reducing body fat percentage [−2.2 (95% CI −3.5 to 0.9) percentage points] compared to aerobic training alone in this population [50]. However, it is important to note that combined training had greater effects on cardiorespiratory fitness when it was added to the program without reducing aerobic exercise volume [0.36 (95% CI 0.05 to 0.68)]. A summary of the effect sizes is presented in Table 3.

Although there has historically been reluctance to prescribe resistance training for individuals with heart failure, evidence now indicates that it can be performed safely and can improve physical function and quality of life in this population [48,51,52]. A 2017 meta-analysis of 10 studies including 240 individuals with chronic heart failure demonstrated that resistance training alone can increase aerobic capacity and quality of life [52]. Similar results were reported in a 2022 meta-analysis, which concluded that resistance training improved aerobic and functional capacity quality of life [51]. The current recommendations highlight that resistance training may be feasible initial rehabilitation strategy for individuals who are deconditioned to the level where aerobic exercise might be difficult [48,53].

### 3.5. Dyslipidemia

Dyslipidemia refers to abnormal levels of lipids, such as low-density lipoproteins, high-density lipoproteins and triglycerides, in the blood stream [54]. Dyslipidemia is a major risk factor for cardiovascular disease [55,56,57,58].

Currently, the role of resistance training in the primary prevention of dyslipidemia is not clear. According to a large meta-analysis of 173 trials, resistance training does not have significant effect on total cholesterol, low-density lipoprotein cholesterol, high-density lipoprotein cholesterol, or triglycerides in a mixed population of younger and older adults [59]. However, their subgroup analysis demonstrated significant improvements in total cholesterol, high-density lipoprotein cholesterol, and triglycerides in over 40-year-olds compared to younger adults [59]. This could indicate that resistance training can be used to prevent dyslipidemia among those who are over 40 years old but more research is needed to establish the association.

**Table 3:** Summary of the results of the meta-analyses on the effect of resistance training interventions on health outcomes among people living with hypertension, cardiovascular disease or dyslipidemia.

Condition	Population	Intervention	Outcome and Effect Size	Reference
Hypertension	Hypertensive adults and older adults	RT	Systolic blood pressure −9.52 (95% CI −12.89 to −6.14) mmHg Diastolic blood pressure −5.19 (95% CI −7.98 to −2.39) mmHg	Correia 2023 [44]
	Prehypertensive and hypertensive adults and older adults		Systolic blood pressure −8.2 (95% CI −10.9 to −5.5) mmHg Diastolic blood pressure −4.1 (95% CI −6.3 to −1.9) mmHg	De Sousa 2017 [43]
Cardiovascular disease	Adults with coronary artery disease	RT	Cardiorespiratory fitness 0.26 (95% CI 0.02 to 0.49)] Lean body mass 0.8 (95% CI 0.4 to 1.2) kg Body fat% −2.2 (95% CI −3.5 to −0.9)	Terada 2024 [50]
	Adults with heart failure		Aerobic capacity 2.64 (95% CI 1.67 to 3.60) mL/kg/min Functional capacity 49.94 (95% CI 34.59 to 65.29) Quality of life − 8.25 (95% CI −11.51 to −4.99)	Fisher 2022 [51]
	Adults and older adults with heart failure		Aerobic capacity 2.71 (95% CI 1.96 to 3.45) mL/kg/min Functional capacity 59.26 (95% CI 36.75 to 18.78) Quality of life − 5.71(95% CI −9.85 to −1.56)	Giuliano 2017 [52]
Dyslipidemia	Postmenopausal women with dyslipidemia	RT	Total cholesterol −12.36 (95% CI −19.64 to −5.08) mg/dL Low-density lipoprotein cholesterol −14.38 (95% CI −23.49 to −5.28) mg/dL	He 2023 [60]

RT = resistance training; CI = confidence interval.

Resistance training might have a role in the treatment of dyslipidemia. Resistance training has been shown to improve lipid profiles among postmenopausal women with dyslipidemia [60]. A meta-analysis of 19 studies demonstrated that resistance training reduced total cholesterol by −12.36 (95% CI −19.64 to −5.08) mg/dL and low-density lipoprotein levels by −14.38 (95% CI −23.49 to −5.28) mg/dL among the postmenopausal women with elevated values at the beginning of the intervention [60]. The effect sizes is presented in Table 3.

In summary, the evidence on the impact of resistance training in the prevention and treatment of dyslipidemia is inconclusive. It has been suggested that the lipoprotein-lipid response to resistance training could be genotype dependent [61], which would explain why some studies and meta-analysis report positive changes and some report no effect. Overall, more evidence is needed regarding the role of resistance training in the prevention and management of dyslipidemia.

### 3.6. Osteoporosis

Osteoporosis is considered to be the most common bone disorder [62]. It is characterized by compromised bone strength, which leads to increased risk of fractures [63]. Bone strength is considered to be a reflection of both bone density and bone quality [64].

Primary prevention of osteoporosis takes place in childhood, adolescence and early adulthood [64,65]. The attainment of maximum peak bone mass during these critical life stages is considered the most effective defense against osteoporosis, given that individuals with higher peak bone mass following adolescence experience greater resistance to subsequent reductions in bone density [64,65]. The maximization of peak bone mass is achieved through a combination of nutrition, loading and exposure to sex hormones during puberty [64,65]. In adulthood, after peak bone mass has been achieved typically by the early twenties, osteoporosis prevention focuses on slowing down the loss of bone mineral density [64,65].

Regarding the treatment of osteoporosis, the nonpharmacological interventions have focused on nutrition and the role of exercise has been fairly limited, leading to limited evidence on the role of resistance training [62,66]. In a 2019 article, Coronado-Zarco and colleagues reviewed six clinical practice guidelines for the nonpharmacological osteoporosis treatment and created 14 recommendations for practitioners [66]. In these recommendations, the role of exercise is limited to the prevention of falls [66]. Similarly, the 2021 position statement of the North American Menopause Society on the management of osteoporosis in postmenopausal women states that there is not enough evidence supporting exercise as an osteogenic therapy in individuals with osteoporosis [62]. In alignment, the 2024 goal-directed osteoporosis treatment position statement focuses on medical interventions and does not provide guidance related to resistance training or general exercise [67].

However, some recent statements and guidelines provide specific recommendations related to resistance training. A 2022 consensus statement from the United Kingdom on physical activity and exercise for osteoporosis recommends that all individuals with osteoporosis participate in progressive resistance training to maintain bone strength [68]. For people with osteoporosis who are frail and/or less able to exercise, physical activity and exercise should be tailored to individual ability and should prioritize the prevention of falls [68]. Overall, the consensus statement promotes a positive approach to exercise so that people with osteoporosis can have the confidence to do more rather than less, as inactivity can lead to reduced physical function. In addition, the 2024 United Kingdom clinical guideline for the prevention and treatment of osteoporosis [63] provides specific recommendations for resistance training, stating that the combination of weight-bearing and resistance training can effectively reduce bone loss in the femoral neck and lumbar spine in post-menopausal women, based on a meta-analyses of 74 studies [69]. The guideline also states that upper body resistance training can increase bone mass, but it is important to note that this is based on a meta-analysis of adults participating in upper limb exercise interventions to improve bone mass and not adults living with osteoporosis [70]. Therefore, this is not evidence about reversal of osteoporosis. While an individual trial, such as the LIFTMOR trial [71] utilizing high-intensity resistance and impact training, reported improvements in indices of bone strength, meta-analytic evidence is still needed. A summary of recommendations from recent guidelines and statements is presented in Table 4.

In summary, the current evidence indicates that exercise does not reverse osteoporosis, resistance training provides important functional benefits, including improved physical performance and a greater ability to perform activities of daily living, such as sitting down and standing up independently [71]. It is important to note that while osteoporosis is not a contraindication to resistance training, there are some safety precautions that need to be taken into consideration in programming. For example, it is important to avoid high degrees of spinal flexion [68], as that can increase the risk of a vertebral compression fracture due to the changes in the shape of the vertebral bodies. An example of an exercise to avoid is abdominal crunch machine at the gym that adds resistance to a spine forward flexion motion. However, when prescribing resistance training, it is important to use a ‘how to’ approach instead of ‘don’t do’, focusing on teaching individuals with osteoporosis how to stay active safely [68].

## 4. Conclusions

This review summarizes current evidence on the role of resistance training in the prevention and treatment of common lifestyle-related chronic conditions: type 2 diabetes, obesity, hypertension, cardiovascular disease, dyslipidemia, and osteoporosis.

Regarding the prevention of chronic conditions, current evidence demonstrates that resistance training can be beneficial in the prevention of type 2 diabetes and cardiovascular disease. The role of resistance training in the prevention of dyslipidemia is currently inconclusive, and more research is needed. Resistance training can potentially be effective in the prevention of obesity and hypertension. However, studies on these conditions combined the target populations for prevention and treatment, and the results cannot be separated. In osteoporosis prevention, resistance training can be utilized to slow down the loss of bone mineral density.

For treatment, evidence supports resistance training or resistance training in combination with aerobic training to improve glycemic control among those living with type 2 diabetes. Resistance training is also beneficial in the treatment of obesity. However, it is important to note that the best intervention will depend on the goal. Lean body mass can be improved without caloric restriction, but for the goal of reducing body mass or body fat percentage, it is much more effective to combine resistance training with caloric restriction. Resistance training can also reduce blood pressure among adults living with hypertension, improve cardiorespiratory fitness indults with coronary artery disease and improve both aerobic and functional capacity in adults with heart failure. For dyslipidemia, evidence is limited. Resistance training has been shown to improve some lipid values in postmenopausal women with dyslipidemia, but more evidence is needed. For osteoporosis treatment, current guidelines recommend progressive resistance training for treatment but acknowledge that it cannot reverse osteoporosis.

The purpose of this narrative review was not to lift up resistance training as the superior tool for prevention or treatment of chronic conditions over other interventions. As has been stated, in many cases, a combination of activity types, and diet when applicable, can be the most effective. The evidence presented here demonstrates that resistance training has a role in the prevention and treatment of several prevalent chronic conditions, and it is a tool that could be utilized more to support people living with chronic conditions. Resistance training yields different benefits than aerobic training, and the combination of both can be recommended.

Future research could explore those living with chronic conditions who already incorporate regular aerobic training to examine what benefits adding resistance training could provide for disease management. In addition, more research is needed on the dose–response. Identifying the minimal effective dose for health benefits would be relevant for providing more specific guidelines in the future.

The fact that for some chronic conditions the greatest benefits in treatment are yielded from the combination of aerobic exercise and resistance training aligns well with the current physical activity guidelines, which recommend both aerobic physical activity and muscle-strengthening activities to everyone [1,2]. While it can be stated on the population level that the benefits of physical activity participation outweigh the risk among those living with chronic conditions [1], it is important for individuals to consult their medical providers before implementing changes to their exercise habits.

While any activity is better than no activity, it is important to identify strategies to increase participation in resistance training, without losing aerobic exercise participation, among adults.

## Figures and Tables

**Table 1:** Summary of the results of the meta-analyses on the effect of exercise intervention on HbA1c among people living with type 2 diabetes.

Condition	Population	Intervention	Outcome and Effect Size	Reference
Type 2 diabetes	Middle-aged and older adults with T2DM	High-intensity RT Moderate-intensity aerobic High-intensity aerobic + moderate-intensity RT Low-intensity RT	HbA_1c_ −0.62 (95% CI −0.93 to −0.30) pp − 0.58 (95% CI −1.10 to −0.05) pp − 0.54 (95% CI −1.02 to −0.06) pp − 0.54 (95% CI −1.00 to −0.09) pp	Yu 2026 [23]
	Adults with T2DM	RT + aerobic High-intensity interval training Continuous aerobic RT	HbA_1c_ −0.74 (95% CI −0.91 to −0.57) pp −0.71 (95% CI −1.07to −0.35) pp −0.62 (95% CI −0.84 to −0.41) pp −0.36 (95% CI −0.51 to −0.20) pp	Michielsen 2025 [22]
	Adults with T2DM	Aerobic RT RT + aerobic	HbA_1c_ −0.73 (95% CI −1.06 to −0.40) pp −0.57 (95% CI −1.14 to −0.01) pp −0.51 (95% CI −0.79 to −0.23) pp	Umpierre 2011 [24]

RT = resistance training; T2DM = type 2 diabetes mellitus; CI = confidence interval; pp = percentage point.

**Table 2:** Summary of the results of the meta-analyses on the effect of exercise and diet interventions on body composition among people with overweight/obesity.

Condition	Population	Intervention	Outcome and Effect Size	Reference
Obesity	Adults 55 to 70 years with overweight/obesity	Energy restriction + high protein RT + energy restriction Energy restriction + high protein + exercise Aerobic + energy restriction Mixed exercise + energy restriction Energy restriction Mixed exercise 5:2 diet RT Aerobic RT + high protein	Total mass −5.86 (95% CI −7.34 to −4.37) kg −5.74 (95% CI −6.91 to −4.57) kg −5.56 (95% CI −7.24 to −3.89) kg −5.00 (95% CI −6.34 to −3.65) kg −4.98 (95% CI −6.33 to −3.64) kg −4.26 (95% CI −5.35 to −3.17) kg −2.26 (95% CI −3.29 to −1.23) kg −1.80 (95% CI −4.43 to 0.83) kg −1.49 (95% CI −2.52 to −0.45) kg −1.29 (95% CI −2.83 to 0.25) kg −1.21 (95% CI −4.01 to 1.58) kg	Eglseer 2023 [34]
	Children and adults with overweight/obesity	RT RT + caloric restriction RT + aerobic RT + aerobic + caloric restriction RT + aerobic + healthy diet	Body fat % −1.6 (95% CI −1.9 to −1.2) pp −3.8 (95% CI −4.7 to −2.9) pp −2.3 (95% CI −2.7 to −1.9) pp −3.0 (95% CI −4.1 to −1.8) pp −2.3 (95% CI −2.8 to −1.8) pp	Lopez 2022 [33]
		RT RT + caloric restriction RT + low-sugar diet RT + protein supplement RT + aerobic RT + aerobic + caloric restriction	Total mass −1.0 (95% CI −1.4 to −0.7) kg −5.1 (95% CI −6.3 to −3.8) kg 0.2 (95% CI −1.7 to 2.0) kg −0.7 (95% CI −3.4 to 2.1) kg −1.4 (95% CI −2.0 to −0.8) kg −5.3 (95% CI −7.2 to −3.5) kg	
		RT RT + caloric restriction RT + low-sugar diet RT + aerobic RT + aerobic + caloric restriction	Lean mass 0.8 (95% CI 0.6 to 1.0) kg −0.2 (95% CI −1.2 to 0.8) kg 1.2 (95% CI −0.4 to 2.7) kg 0.6 (95% CI 0.3 to 0.9) kg −0.3 (95% CI −1.4 to 0.8) kg	

RT = resistance training; CI = confidence interval; pp = percentage point.

**Table 4:** Summary of the recommendations related to resistance training for people living with osteoporosis from recent clinical guidelines and consensus and position statements.

Group/ Medical Society	Country and Year	Recommendation on Resistance Training	Reference
National Osteoporosis Guideline Group	United Kingdom 2024	Effect of exercise on skeletal sites varies. Combination of weight-bearing and resistance strengthening exercise can be effective in reducing bone loss in the femoral neck and lumbar spine in post-menopausal women. Upper body resistance training can increase bone mass in the forearm. Resistance training can be included in combined exercise protocols to prevent falls.	Gregson 2025 [63] The 2024 UK clinical guideline for the prevention and treatment of osteoporosis
American Society for Bone and Mineral Research and Bone Health & Osteoporosis Foundation task force	United States 2024	No recommendation	Cosman 2024 [67] Goal-directed osteoporosis treatment: ASBMR/BHOF task force position statement 2024
UK Expert Exercise Steering Group	United Kingdom 2022	Muscle-strengthening activities are recommended 2–3 days a week to maintain bone strength. For maximum benefit, muscle-strengthening should include progressive resistance training. All muscle groups should be targeted. For people with osteoporosis who are frail and/or less able to exercise, physical activity/exercise should be adapted to their abilities.	Brooke-Wavell 2022 [68] Strong, steady and straight: UK consensus statement on physical activity and exercise for osteoporosis
North American Menopause Society	United States 2021	The perception that exercise can induce new bone formation in postmenopausal women with osteoporosis is unfounded. Programs of regular exercise for general health can be recommended. Women with vertebral fractures should avoid activities that involve lifting or pulling with forward spine flexion or rotation	[62] Management of osteoporosis in postmenopausal women: the 2021 position statement of The North American Menopause Society

## Data Availability

No new data were created or analyzed in this study. Data sharing is not applicable to this article.

## Abbreviations

The following abbreviations are used in this manuscript:

CI	confidence interval
HbA_1c_	Hemoglobin A_1c_

## References

[1] Bull F.C., Al-Ansari S.S., Biddle S., Borodulin K., Buman M.P., Cardon G., Carty C., Chaput J.-P., Chastin S., Chou R. (2020). World Health Organization 2020 Guidelines on Physical Activity and Sedentary Behaviour. Br. J. Sports Med..

[2] Piercy K.L., Troiano R.P., Ballard R.M., Carlson S.A., Fulton J.E., Galuska D.A., George S.M., Olson R.D. (2018). The Physical Activity Guidelines for Americans. J. Am. Med. Assoc..

[3] World Health Organization (2021). Health Promotion Glossary of Terms 2021.

[4] Ferrari A.J., Santomauro D.F., Aali A., Abate Y.H., Abbafati C., Abbastabar H., ElHafeez S.A., Abdelmasseh M., Abd-Elsalam S., Abdollahi A. (2024). Global Incidence, Prevalence, Years Lived with Disability (YLDs), Disability-Adjusted Life-Years (DALYs), and Healthy Life Expectancy (HALE) for 371 Diseases and Injuries in 204 Countries and Territories and 811 Subnational Locations, 1990–2021: A Systematic Analysis for the Global Burden of Disease Study 2021. Lancet.

[5] Naghavi M., Ong K.L., Aali A., Ababneh H.S., Abate Y.H., Abbafati C., Abbasgholizadeh R., Abbasian M., Abbasi-Kangevari M., Abbastabar H. (2024). Global Burden of 288 Causes of Death and Life Expectancy Decomposition in 204 Countries and Territories and 811 Subnational Locations, 1990–2021: A Systematic Analysis for the Global Burden of Disease Study 2021. Lancet.

[6] Vos T., Abajobir A.A., Abbafati C., Abbas K.M., Abate K.H., Abd-Allah F., Abdulle A.M., Abebo T.A., Abera S.F., Aboyans V. (2017). Global, Regional, and National Incidence, Prevalence, and Years Lived with Disability for 328 Diseases and Injuries for 195 Countries, 1990-2016: A Systematic Analysis for the Global Burden of Disease Study 2016. Lancet.

[7] Agborsangaya C.B., Lau D., Lahtinen M., Cooke T., Johnson J.A. (2013). Health-Related Quality of Life and Healthcare Utilization in Multimorbidity: Results of a Cross-Sectional Survey. Qual. Life Res..

[8] Zhang Y., Li Y., Peila R., Wang T., Xue X., Kaplan R.C., Dannenberg A.J., Qi Q., Rohan T.E. (2024). Associations of Lifestyle and Genetic Risks with Obesity and Related Chronic Diseases in the UK Biobank: A Prospective Cohort Study. Am. J. Clin. Nutr..

[9] Wehby G.L., Domingue B.W., Wolinsky F.D. (2018). Genetic Risks for Chronic Conditions: Implications for Long-Term Wellbeing. J. Gerontol.-Ser. A Biol. Sci. Med. Sci..

[10] Momma H., Kawakami R., Honda T., Sawada S.S. (2022). Muscle-Strengthening Activities Are Associated with Lower Risk and Mortality in Major Non-Communicable Diseases: A Systematic Review and Meta-Analysis of Cohort Studies. Br. J. Sports Med..

[11] Elgaddal N., Kramarow E.A., Reuben C. (2020). Physical Activity Among Adults Aged 18 and Over: United States, 2020.

[12] Currier B.S., D’Souza A.C., Singh M.A.F., Lowisz C.V., Rawson E.S., Schoenfeld B.J., Smith-Ryan A.E., Steen J.P., Thomas G.A., Triplett N.T. (2026). American College of Sports Medicine Position Stand. Resistance Training Prescription for Muscle Function, Hypertrophy, and Physical Performance in Healthy Adults: An Overview of Reviews. Med. Sci. Sports Exerc..

[13] Ratamess N., Alvar B., Evetoch T., Housh T., Kibler W.B., Kraemer W.J., Triplett N.T. (2009). Progression Models in Resistance Training for Healthy Adults. Med. Sci. Sports Exerc..

[14] Kraemer W.J., Ratamess N.A. (2004). Fundamentals of Resistance Training: Progression and Exercise Prescription. Med. Sci. Sports Exerc..

[15] Khan M.A.B., Hashim M.J., King J.K., Govender R.D., Mustafa H., Kaabi J. (2020). Al Epidemiology of Type 2 Diabetes—Global Burden of Disease and Forecasted Trends. J. Epidemiol. Glob. Health.

[16] American Diabetes Association Professional Practice Committee for Diabetes 1 (2026). Improving Care and Promoting Health in Populations: Standards of Care in Diabetes—2026. Diabetes Care.

[17] American Diabetes Association Professional Practice Committee for Diabetes 2 (2026). Diagnosis and Classification of Diabetes: Standards of Care in Diabetes-2026. Diabetes Care.

[18] Alberti K.G.M.M., Zimmet P.Z. (1998). Definition, Diagnosis and Classification of Diabetes Mellitus and Its Complications. Part 1: Diagnosis and Classification of Diabetes Mellitus. Provisional Report of a WHO Consultation. Diabet. Med..

[19] Grøntved A., Rimm E.B., Willett W.C., Andersen L.B., Hu F.B. (2012). A Prospective Study of Weight Training and Risk of Type 2 Diabetes Mellitus in Men. Arch. Intern. Med..

[20] Grøntved A., Pan A., Mekary R.A., Stampfer M., Willett W.C., Manson J.A.E., Hu F.B. (2014). Muscle-Strengthening and Conditioning Activities and Risk of Type 2 Diabetes: A Prospective Study in Two Cohorts of US Women. PLoS Med..

[21] Shiroma E.J., Cook N.R., Manson J.E., Moorthy M., Buring J.E., Rimm E.B., Lee I.M. (2017). Strength Training and the Risk of Type 2 Diabetes and Cardiovascular Disease. Med. Sci. Sports Exerc..

[22] Michielsen M., Yagiz J., Hanssens M., Claes J., Geuns L., Gojevic T., Hansen D., Claessen G., De Craemer M., Cornelissen V. (2025). The Effect of Exercise Characteristics on HbA1c and Other Cardiovascular Risk Factors in Adults with Type 2 Diabetes: A Systematic Review and Meta-Analysis of Randomised Controlled Trials. Cardiovasc. Diabetol..

[23] Yu J., Li X., Yu H., Huang Y. (2026). Comparative Effects of Different Intensities of Aerobic and Resistance Exercise on Glycemic Control and Cardiorespiratory Fitness in Middle-Aged Older Patients with Type 2 Diabetes: A Network Meta-Analysis. Front. Public Health.

[24] Umpierre D., Ribeiro P.A.B., Kramer C.K., Leitão C.B., Zucatti A.T.N., Azevedo M.J., Gross J.L., Ribeiro J.P., Schaan B.D. (2011). Physical Activity Advice Only or Structured Exercise Training and Association with HbA1c Levels in Type 2 Diabetes: A Systematic Review and Meta-Analysis. JAMA.

[25] Jansson A.K., Chan L.X., Lubans D.R., Duncan M.J., Plotnikoff R.C. (2022). Effect of Resistance Training on HbA1c in Adults with Type 2 Diabetes Mellitus and the Moderating Effect of Changes in Muscular Strength: A Systematic Review and Meta-Analysis. BMJ Open Diabetes Res. Care.

[26] Sigal R.J., Boulé N.G., Anthony Wells G., Kenny G.P., Wells G.A., Prud D., Fortier M., Reid R.D., Tulloch H., Coyle D. (2007). Effects of Aerobic Training, Resistance Training, or Both on Glycemic Control in Type 2 Diabetes-A Randomized Trial Effects of Aerobic Training, Resistance Training, or Both on Glycemic Control in Type 2 Diabetes A Randomized Trial. Artic. Ann. Intern. Med..

[27] Church T.S., Blair S.N., Shannon Cocreham P., Neil Johannsen B., Johnson W., Kramer K., Catherine Mikus M.R., Valerie Myers M., Nauta M., Ruben Rodarte B.Q. (2010). Effects of Aerobic and Resistance Training on Hemoglobin A 1c Levels in Patients With Type 2 Diabetes A Randomized Controlled Trial. JAMA.

[28] Busetto L., Dicker D., Frühbeck G., Halford J.C.G., Sbraccia P., Yumuk V., Goossens G.H. (2024). A New Framework for the Diagnosis, Staging and Management of Obesity in Adults. Nat. Med..

[29] Bray G.A., Frühbeck G., Ryan D.H., Wilding J.P.H. (2016). Management of Obesity. Lancet.

[30] Oppert J.M., Bellicha A., van Baak M.A., Battista F., Beaulieu K., Blundell J.E., Carraça E.V., Encantado J., Ermolao A., Pramono A. (2021). Exercise Training in the Management of Overweight and Obesity in Adults: Synthesis of the Evidence and Recommendations from the European Association for the Study of Obesity Physical Activity Working Group. Obes. Rev..

[31] Wharton S., Lau D.C.W., Vallis M., Sharma A.M., Biertho L., Campbell-Scherer D., Adamo K., Alberga A., Bell R., Boulé N. (2020). Obesity in Adults: A Clinical Practice Guideline. Can. Med. Assoc. J..

[32] Nadolsky K., Garvey W.T., Agarwal M., Bonnecaze A., Burguera B., Chaplin M.D.G., Griebeler M.L., Harris S.R., Schellinger J.N., Simonetti J. (2025). American Association of Clinical Endocrinology Consensus Statement: Algorithm for the Evaluation and Treatment of Adults with Obesity/Adiposity-Based Chronic Disease—2025 Update. Endocr. Pract..

[33] Lopez P., Taaffe D.R., Galvão D.A., Newton R.U., Nonemacher E.R., Wendt V.M., Bassanesi R.N., Turella D.J.P., Rech A. (2022). Resistance Training Effectiveness on Body Composition and Body Weight Outcomes in Individuals with Overweight and Obesity across the Lifespan: A Systematic Review and Meta-Analysis. Obes. Rev..

[34] Eglseer D., Traxler M., Embacher S., Reiter L., Schoufour J.D., Weijs P.J.M., Voortman T., Boirie Y., Cruz-Jentoft A., Bauer S. (2023). Nutrition and Exercise Interventions to Improve Body Composition for Persons with Overweight or Obesity Near Retirement Age: A Systematic Review and Network Meta-Analysis of Randomized Controlled Trials. Adv. Nutr..

[35] Morze J., Rücker G., Danielewicz A., Przybyłowicz K., Neuenschwander M., Schlesinger S., Schwingshackl L. (2021). Impact of Different Training Modalities on Anthropometric Outcomes in Patients with Obesity: A Systematic Review and Network Meta-Analysis. Obes. Rev..

[36] Lopez P., Radaelli R., Taaffe D.R., Galvaõ D.A., Newton R.U., Nonemacher E.R., Wendt V.M., Bassanesi R.N., Turella D.J.P., Rech A. (2022). Moderators of Resistance Training Effects in Overweight and Obese Adults: A Systematic Review and Meta-Analysis. Med. Sci. Sports Exerc..

[37] Orange S.T., Madden L.A., Vince R.V. (2020). Resistance Training Leads to Large Improvements in Strength and Moderate Improvements in Physical Function in Adults Who Are Overweight or Obese: A Systematic Review. J. Physiother..

[38] Davis M.E., Blake C., Perrotta C., Cunningham C., O’Donoghue G. (2022). Impact of Training Modes on Fitness and Body Composition in Women with Obesity: A Systematic Review and Meta-Analysis. Obesity.

[39] Byrne H.K., Wilmore H. (2001). The Effects of a 20-Week Exercise Training Program on Resting Metabolic Rate in Previously Sedentary, Moderately Obese Women. Int. J. Sport Nutr. Exerc. Metab..

[40] American Heart Association Understanding Blood Pressure Readings. https://www.heart.org/en/health-topics/high-blood-pressure/understanding-blood-pressure-readings.

[41] Kelley G.A., Kelley K.S. (2000). Progressive Resistance Exercise and Resting Blood Pressure A Meta-Analysis of Randomized Controlled Trials. Hypertension.

[42] Jabbarzadeh Ganjeh B., Zeraattalab-Motlagh S., Jayedi A., Daneshvar M., Gohari Z., Norouziasl R., Ghaemi S., Selk-Ghaffari M., Moghadam N., Kordi R. (2024). Effects of Aerobic Exercise on Blood Pressure in Patients with Hypertension: A Systematic Review and Dose-Response Meta-Analysis of Randomized Trials. Hypertens. Res..

[43] De Sousa E.C., Abrahin O., Ferreira A.L.L., Rodrigues R.P., Alves E.A.C., Vieira R.P. (2017). Resistance Training Alone Reduces Systolic and Diastolic Blood Pressure in Prehypertensive and Hypertensive Individuals: Meta-Analysis. Hypertens. Res..

[44] Correia R.R., Veras A.S.C., Tebar W.R., Rufino J.C., Batista V.R.G., Teixeira G.R. (2023). Strength Training for Arterial Hypertension Treatment: A Systematic Review and Meta-Analysis of Randomized Clinical Trials. Sci. Rep..

[45] Pescatello L.S., Buchner D.M., Jakicic J.M., Powell K.E., Kraus W.E., Bloodgood B., Campbell W.W., Dietz S., Dipietro L., George S.M. (2019). Physical Activity to Prevent and Treat Hypertension: A Systematic Review. Med. Sci. Sports Exerc..

[46] Sharman J.E., La Gerche A., Coombes J.S. (2015). Exercise and Cardiovascular Risk in Patients with Hypertension. Am. J. Hypertens..

[47] World Health Organization Cardiovascular Diseases (CVDs). https://www.who.int/news-room/fact-sheets/detail/cardiovascular-diseases-(cvds).

[48] Paluch A.E., Boyer W.R., Franklin B.A., Laddu D., Lobelo F., Lee D.C., Mcdermott M.M., Swift D.L., Webel A.R., Lane A. (2024). Resistance Exercise Training in Individuals With and Without Cardiovascular Disease: 2023 Update: A Scientific Statement From the American Heart Association. Circulation.

[49] Kokkinos P., Faselis C., Samuel I.B.H., Lavie C.J., Zhang J., Vargas J.D., Pittaras A., Doumas M., Karasik P., Moore H. (2023). Changes in Cardiorespiratory Fitness and Survival in Patients With or Without Cardiovascular Disease. J. Am. Coll. Cardiol..

[50] Terada T., Pap R., Thomas A., Wei R., Noda T., Visintini S., Reed J.L. (2024). Effects of Muscle Strength Training Combined with Aerobic Training versus Aerobic Training Alone on Cardiovascular Disease Risk Indicators in Patients with Coronary Artery Disease: A Systematic Review and Meta-Analysis of Randomised Clinical Trials. Br. J. Sports Med..

[51] Fisher S., Smart N.A., Pearson M.J. (2022). Resistance Training in Heart Failure Patients: A Systematic Review and Meta-Analysis. Heart Fail. Rev..

[52] Giuliano C., Karahalios A., Neil C., Allen J., Levinger I. (2017). The Effects of Resistance Training on Muscle Strength, Quality of Life and Aerobic Capacity in Patients with Chronic Heart Failure—A Meta-Analysis. Int. J. Cardiol..

[53] Conraads V.M., Beckers P.J. (2010). Exercise Training in Heart Failure: Practical Guidance. Heart.

[54] Pappan N., Awosika A., Rehman A. (2024). Dyslipidemia. StatPearls [Internet].

[55] Du Z., Qin Y. (2023). Dyslipidemia and Cardiovascular Disease: Current Knowledge, Existing Challenges, and New Opportunities for Management Strategies. J. Clin. Med..

[56] Budoff M. (2016). Triglycerides and Triglyceride-Rich Lipoproteins in the Causal Pathway of Cardiovascular Disease. Am. J. Cardiol..

[57] Nordestgaard B.G. (2016). Triglyceride-Rich Lipoproteins and Atherosclerotic Cardiovascular Disease: New Insights from Epidemiology, Genetics, and Biology. Circ. Res..

[58] Chapman M.J., Ginsberg H.N., Amarenco P., Andreotti F., Borén J., Catapano A.L., Descamps O.S., Fisher E., Kovanen P.T., Kuivenhoven J.A. (2011). Triglyceride-Rich Lipoproteins and High-Density Lipoprotein Cholesterol in Patients at High Risk of Cardiovascular Disease: Evidence and Guidance for Management. Eur. Heart J..

[59] Ashton R.E., Tew G.A., Aning J.J., Gilbert S.E., Lewis L., Saxton J.M. (2020). Effects of Short-Term, Medium-Term and Long-Term Resistance Exercise Training on Cardiometabolic Health Outcomes in Adults: Systematic Review with Meta-Analysis. Br. J. Sports Med..

[60] He M., Hu S., Wang J., Wang J., Găman M.A., Hariri Z., Tian Y. (2023). Effect of Resistance Training on Lipid Profile in Postmenopausal Women: A Systematic Review and Meta-Analysis of Randomized Controlled Trials. Eur. J. Obstet. Gynecol. Reprod. Biol..

[61] Hurley B.F., Hanson E.D., Sheaff A.K. (2011). Strength Training as a Countermeasure to Aging Muscle and Chronic Disease. Sports Med..

[62] (2021). Management of Osteoporosis in Postmenopausal Women: The 2021 Position Statement of The North American Menopause Society. Menopause.

[63] Gregson C.L., Armstrong D.J., Avgerinou C., Bowden J., Cooper C., Douglas L., Edwards J., Gittoes N.J.L., Harvey N.C., Kanis J.A. (2025). The 2024 UK Clinical Guideline for the Prevention and Treatment of Osteoporosis. Arch. Osteoporos..

[64] (2001). NIH Consensus Development Panel on Osteoporosis Prevention, D. and T. Osteoporosis Prevention, Diagnosis, and Therapy. JAMA.

[65] Heaney R.P., Abrams S., Dawson-Hughes B., Looker A., Marcus R., Matkovic V., Weaver C. (2000). Peak Bone Mass. Osteoporos. Int..

[66] Coronado-Zarco R., Olascoaga-Gómez de León A., García-Lara A., Quinzaños-Fresnedo J., Nava-Bringas T.I., Macías-Hernández S.I. (2019). Nonpharmacological Interventions for Osteoporosis Treatment: Systematic Review of Clinical Practice Guidelines. Osteoporos. Sarcopenia.

[67] Cosman F., Lewiecki E.M., Eastell R., Ebeling P.R., Jan De Beur S., Langdahl B., Rhee Y., Fuleihan G.E.H., Kiel D.P., Schousboe J.T. (2024). Goal-Directed Osteoporosis Treatment: ASBMR/BHOF Task Force Position Statement 2024. J. Bone Miner. Res..

[68] Brooke-Wavell K., Skelton D.A., Barker K.L., Clark E.M., De Biase S., Arnold S., Paskins Z., Robinson K.R., Lewis R.M., Tobias J.H. (2022). Strong, Steady and Straight: UK Consensus Statement on Physical Activity and Exercise for Osteoporosis. Br. J. Sports Med..

[69] Kemmler W., Shojaa M., Kohl M., von Stengel S. (2020). Effects of Different Types of Exercise on Bone Mineral Density in Postmenopausal Women: A Systematic Review and Meta-Analysis. Calcif. Tissue Int..

[70] Babatunde O.O., Bourton A.L., Hind K., Paskins Z., Forsyth J.J. (2020). Exercise Interventions for Preventing and Treating Low Bone Mass in the Forearm: A Systematic Review and Meta-Analysis. Arch. Phys. Med. Rehabil..

[71] Watson S.L., Weeks B.K., Weis L.J., Harding A.T., Horan S.A., Beck B.R. (2018). High-Intensity Resistance and Impact Training Improves Bone Mineral Density and Physical Function in Postmenopausal Women With Osteopenia and Osteoporosis: The LIFTMOR Randomized Controlled Trial. J. Bone Miner. Res..

